# Low Quantitative Blush Evaluator score predicts larger infarct size and reduced left ventricular systolic function in patients with STEMI regardless of diabetes status

**DOI:** 10.1038/s41598-022-24855-6

**Published:** 2023-01-05

**Authors:** Katarzyna Nabrdalik, Andrzej Tomasik, Krzysztof Irlik, Mirela Hendel, Hanna Kwiendacz, Edyta Radzik, Katarzyna Pigoń, Tomasz Młyńczak, Janusz Gumprecht, Ewa Nowalany-Kozielska, Gregory Y. H. Lip

**Affiliations:** 1 Department of Internal Medicine, Diabetology and Nephrology, Faculty of Medical Sciences in Zabrze, Medical University of Silesia, Katowice, Poland; 2 Liverpool Centre for Cardiovascular Science, University of Liverpool, Liverpool John Moores University and Liverpool Heart & Chest Hospital, Liverpool, UK; 3 Students’ Scientific Association By the Department of Internal Medicine, Diabetology and Nephrology, Faculty of Medical Sciences in Zabrze, Medical University of Silesia, Katowice, Poland; 4 2nd Department of Cardiology in Zabrze, Faculty of Medical Sciences in Zabrze, Medical University of Silesia, Katowice, Poland; 5 Department of Clinical Medicine, Aalborg University, Aalborg, Denmark

**Keywords:** Cardiology, Endocrine system and metabolic diseases

## Abstract

Type 2 diabetes mellitus (T2DM) and diminished myocardial perfusion increase the risk of heart failure (HF) and/or all-cause mortality during 6-year follow up following primary percutaneous coronary intervention (pPCI) for ST elevation myocardial infarction (STEMI). The aim of the present study was to evaluate the impact of myocardial perfusion on infarct size and left ventricular ejection fraction (LVEF) in patients with T2DM and STEMI treated with pPCI. This is an ancillary analysis of an observational cohort study of T2DM patients with STEMI. We enrolled 406 patients with STEMI, including 104 with T2DM. Myocardial perfusion was assessed with the Quantitative Myocardial Blush Evaluator (QUBE) and infarct size with the creatine kinase myocardial band (CK-MB) maximal activity and troponin area under the curve. LVEF was measured with biplane echocardiography using Simpson’s method at admission and hospital discharge. Analysis of covariance was used for modeling the association between myocardial perfusion, infarct size and left ventricular systolic function. Patients with T2DM and diminished perfusion (QUBE below median) had the highest CK-MB maximal activity (252.7 ± 307.2 IU/L, P < 0.01) along with the lowest LVEF (40.6 ± 10.0, P < 0.001). Older age (p = 0.001), QuBE below median (p = 0.026), and maximal CK-MB activity (p < 0.001) were independent predictors of LVEF. Diminished myocardial perfusion assessed by QuBE predicts significantly larger enzymatic infarct size and lower LVEF among patients with STEMI treated with pPCI, regardless of diabetes status.

## Introduction

Diabetes mellitus remains an important risk factor for worse prognosis in patients after acute ST-segment-elevation myocardial infarction (STEMI) treated with primary percutaneous coronary intervention (pPCI)^1–4^. Although pPCI is the optimal choice of reperfusion therapy in such conditions^5,6^, even timely performed procedures resulting in recanalization of the culprit vessel may still yield disappointing results. This phenomenon is thought to be related to impaired myocardial perfusion^7–10^.

Type 2 diabetes mellitus (T2DM) is associated with diminished myocardial perfusion after reperfusion^11–13^, and even otherwise healthy patients with T2DM exhibit myocardial perfusion defects^14^. Therefore, it is important to establish how this pathology translates into patient clinical outcomes.

We have previously reported that T2DM and diminished myocardial perfusion increased the clinical risks of heart failure (HF) and/or all-cause mortality during 6-year follow-up following pPCI for STEMI^15^. In our study it was also noted that patients with diabetes had worse myocardial perfusion or epicardial flow presented with larger enzymatic infarct size and lower left ventricular ejection fraction (LVEF) when compared to patients without T2DM^15^. Indeed, LVEF is a potent and most commonly used functional marker of the severity of the underlying myocardial injury^16^, yet another robust predictor of adverse events following STEMI is infarct size^17^.

The aim of the study was to evaluate the impact of myocardial perfusion on infarct size and LVEF in patients with T2DM and STEMI treated with pPCI. We tested the hypothesis that patients with T2DM and STEMI treated with pPCI with diminished myocardial perfusion, would have significantly larger enzymatic infarct size and lower LVEF.

## Methods

This is an ancillary analysis of a single center, retrospective, cohort study of patients treated with pPCI due to STEMI. The main results for the clinical outcomes have been published^15^. In brief, we have reviewed 1469 consecutive STEMI patients, who were admitted to cardiology ward from January 2004 until December 2014, out of them 406 patients have fulfilled the following inclusion criteria: age ≥ 18 years old, complete hospital medical records, good quality electrocardiographic tracings and angiograms, and the absence of HF before the hospital admission. Exclusion criteria comprised of coronary artery lesions not amenable to stent implantation or balloon angioplasty, chronic total coronary occlusion which could not be revascularized or referral of the patient for bypass surgery.

Every patient signed a suitable informed consent for in-hospital treatment on admission and no additional consent related to this analysis was necessary since only anonymized registry data has been analyzed. The study protocol has been approved by the Medical University of Silesia Ethics Committee (No. PCN/0022/KB/92/20) and the need for informed consent was waived by this Ethics Committee. All methods were performed in accordance with relevant regulations and the study was conducted in accordance with the Declaration of Helsinki.

Myocardial perfusion has been measured in patients' angiograms with QuBE value using an on-line software available at http://qube.sourceforge.net/. The code for QuBE software is publicly available at https://github.com/mathijs81/qube and has been developed by the University Medical Center Groningen, the Netherlands and thoroughly described by Vogelzang et al.^18^. In brief, the method involves operator-independent, digital analysis of angiogram and calculation of myocardial perfusion score (the higher score reflects better myocardial perfusion). In order to check intra- and inter-rater reliability, random sample of angiograms were analyzed twice by each of the two observers. The inter- and intra-patient variability of QuBE values was 94.5% and 99.7%, respectively. This analysis was performed on 34 angiograms^19^.

Infarct size was assessed by peak activity of creatine kinase myocardial band isoenzyme (CK-MB) and troponin T concentration area under the curve (AUC), measured on hospital admission and 12, 24 and 72 h following admission. Echocardiographic measurement of LVEF was performed on the day of admission and hospital discharge. Detailed information from angiographic reanalysis, angiographic assessment of myocardial perfusion, assessment of infarct size, echocardiographic measurements as well as overview of methods used to obtain biochemical data have been reported previously^19^.

Descriptive statistics were calculated for all the variables, including continuous variables (reported as mean values and standard deviations) and categorical variables (reported as numbers and percentages). The QuBE variable as a binary one was used to stratify patients into groups 1 to 4, according to its median value, then as a continuous variable was used for further analyses. Comparisons were performed using t tests, χ^2^ tests with Yates correction. The one-way or Kruskal ANOVA were used for multiple groups comparisons. Any significance detected with ANOVA was reassessed with χ^2^ or t test with Bonferroni correction for categorical or continuous variables where appropriate. Analysis of covariance was used to adjust for confounding variables. We considered variable as a confounding one if differ significantly among the groups 1 to 4 presented in Tables 1 and 2. All the statistical analyses were performed using Statistica 13 (Statsoft Inc., Tulsa, Oklahoma, USA), and p values < 0.05 were considered to be statistically significant.

### Ethics approval and consent to participate

The study protocol has been approved by the Medical University of Silesia Ethics Committee (No. PCN/0022/KB/92/20).

## Results

Baseline characteristics of the 406 patients (mean age 62.1 ± 10.9 years, 32.8% female) according to QuBE values and presence of T2DM are shown in Table 1. The study population was divided into four groups based on the median QuBE value (9 arbitrary units) and T2DM diagnosis. Differences in age and sex between the groups were significant (p < 0.001, p < 0.01, respectively) whereby patients with T2DM and diminished perfusion were significantly older (p < 0.001), and had the lowest eGFR (p < 0.001). Frequency of previous MI (myocardial infarction) and location of current MI (anterior or inferior) did not differ between groups.

**Table 1:** Baseline characteristics of nondiabetic and diabetic patients, stratified by median QuBE value.

	Nondiabetic		Diabetic		Significance (Kruskal–Wallis)
	QuBE below median (n = 141) (group 1)	QuBE equal or above median (n = 161) (group 2)	QuBE below median (n = 60) (group 3)	QuBE equal or above median (n = 44) (group 4)	
Age, years (X ± SD)	61.9 ± 10.9	59.4 ± 10.9	68.0 ± 9.0	64.9 ± 10.0	P < 0.001^#^
Female, n (%)	42 (29.7)	41 (25.5)	28 (46.7)	22 (50.0)	P < 0.01^##^
Pain duration, min (X ± SD)	362 ± 425	330 ± 318	394 ± 415	361 ± 241	P = 0.823
Hypertension, n (%)	80 (56.7)	59 (36.6)	54 (90.0)	37 (84.0)	P < 0.001^###^
Previous MI, n (%)	23 (16.3)	24 (14.9)	15 (25.0)	7 (15.9)	P = 0.364
Current smokers, n (%)	86 (61.0)	97 (60.2)	17 (28.3)	20 (45.4)	P < 0.001*
eGFR, ml/min/m^2^ (X ± SD)	93.8 ± 26.5	91.3 ± 24.0	78.5 ± 23.7	87.8 ± 26.5	P < 0.01**
Anterior MI, n (%)	60 (42.6)	63 (39.1)	34 (56.7)	17 (38.6)	P = 0.473

*QuBE* quantitative myocardial blush evaluator, *eGFR* estimated glomerular filtration rate, *MI* myocardial infarction.

^#^(1) vs (4) p < 0.001; (1) vs (3) p = 0.001.

^##^(1) vs (3) p = 0.023; (1) vs (4) p = 0.033; (2) vs (4) p = 0.003; (2) vs (3) p = 0.018.

^###^(1) vs (4) p = 0.002; (1) vs (3) p < 0.001; (2) vs (4) p = 0.009; (2) vs (3) p < 0.001.

*(1) vs (3) p < 0.001; (2) vs (3) p < 0.001.

**(2) vs (3) p = 0.005; (1) vs (3) p < 0.001.

Subjects with T2DM and diminished myocardial perfusion had the highest peak CK-MB levels and worst mean LVEF, despite similar troponin T AUC and cTFC (corrected TIMI frame count) levels (Table 2).

**Table 2:** Angiographic, biochemical, echocardiographic and clinical outcomes stratified by diabetes and QuBE.

	Nondiabetic		Diabetic		Significance (Kruskal–Wallis)
	QuBE below median (n = 141) (group 1)	QuBE equal or above median (n = 161) (group 2)	QuBE below median (n = 60) (group 3)	QuBE equal or above median (n = 44) (group 4)	
cTFC, fps (X ± SD)	39.2 ± 27.1	25.4 ± 13.3	38.6 ± 28.7	25.2 ± 15.3	P < 0.01^#^
CK-MB max, IU/L (X ± SD)	240.9 ± 196.8	203.9 ± 224.0	252.7 ± 307.2	149.5 ± 173.6	P < 0.01^##^
Troponin AUC, ng/L × 24 h (X ± SD)	8850 ± 3888	8692 ± 4645	9493 ± 4828	8702 ± 4536	P = 0.492
LVEF (%) (X ± SD)	43.1 ± 10.2	46.2 ± 9.4	40,6 ± 10.0	46.5 ± 11.6	P < 0.001^###^

*AUC* area under the curve, *QuBE* quantitative myocardial blush evaluator, *cTFC* corrected Thrombolysis in Myocardial Infarction frame count, *CK-MB* muscle-brain creatine kinase isoenzyme, *LVEF* left ventricle ejection fraction, *fps* frames per second.

^#^(1) vs (2) p < 0.001; (1) vs (4) p < 0.001.

^##^(1) vs (4) p = 0.02.

^###^(1) vs (2) p = 0.035; (2) vs (3) p = 0.001; (3) vs (4) p = 0.017.

Considering the subgroup of patients with T2DM, those with QuBE score below median had worse procedural outcomes in comparison to patients with higher QuBE score: LVEF was lower (p = 0.017), and peak CK-MB was greater (p = 0.02), but there were no significant differences in troponin T AUC and epicardial flow in infarct-related artery (number of cTFC). There were significantly fewer patients with hypertension and smokers (Table 1). The duration of diabetes was 6.4 ± 4.1 years, all patients were treated with oral hypoglycemic drugs, and 12 patients were treated with insulin. T2DM patients from group 3 and group 4 did not differ in relation to diabetes duration, nor the type of diabetes treatment.

In analysis of covariance, older age (p = 0.001), QuBE below median (p = 0.026), and maximal CK-MB activity (p < 0.001) were independent predictors of LVEF (Table 3).

**Table 3:** Predictors of left ventricle ejection fraction (analysis of covariance).

	Effect	Wald statistics	p
Age	− 0.004	10.505	P < 0.001
Female	− 0.006	0.059	P = 0.808
QuBE	0.005	4.938	P = 0.026
CK-MB max	− 0.0004	43.965	P < 0.001
eGFR	0.0005	1.223	P = 0.269
cTFC	0.0003	1.578	P = 0.345
T2DM	− 0.019	0.490	P = 0.484
Hypertension	0.034	2.122	P = 0.145
Current smoker	0.012	0.287	P = 0.592

*CK-MB* muscle-brain creatine kinase isoenzyme, *QuBE* quantitative myocardial blush evaluator, *T2DM* type 2 diabetes mellitus, *eGFR* estimated glomerular filtration rate.

**Figure 1:**
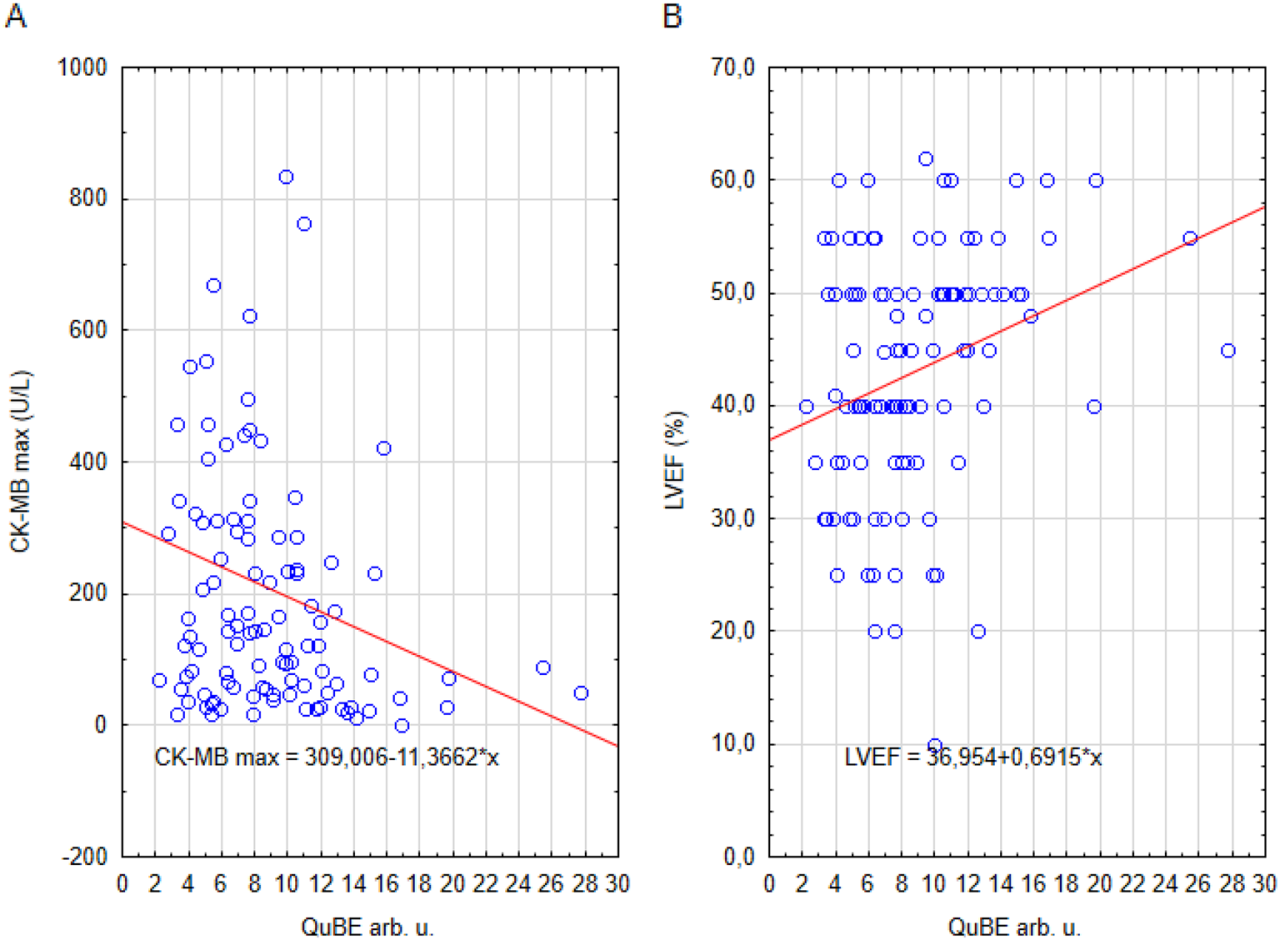
Graphical presentation of the relationship between peak CK-MB activity and QuBE (**A**) as well as LVEF and QuBE (**B**) in T2DM patients.

**Figure 2:**
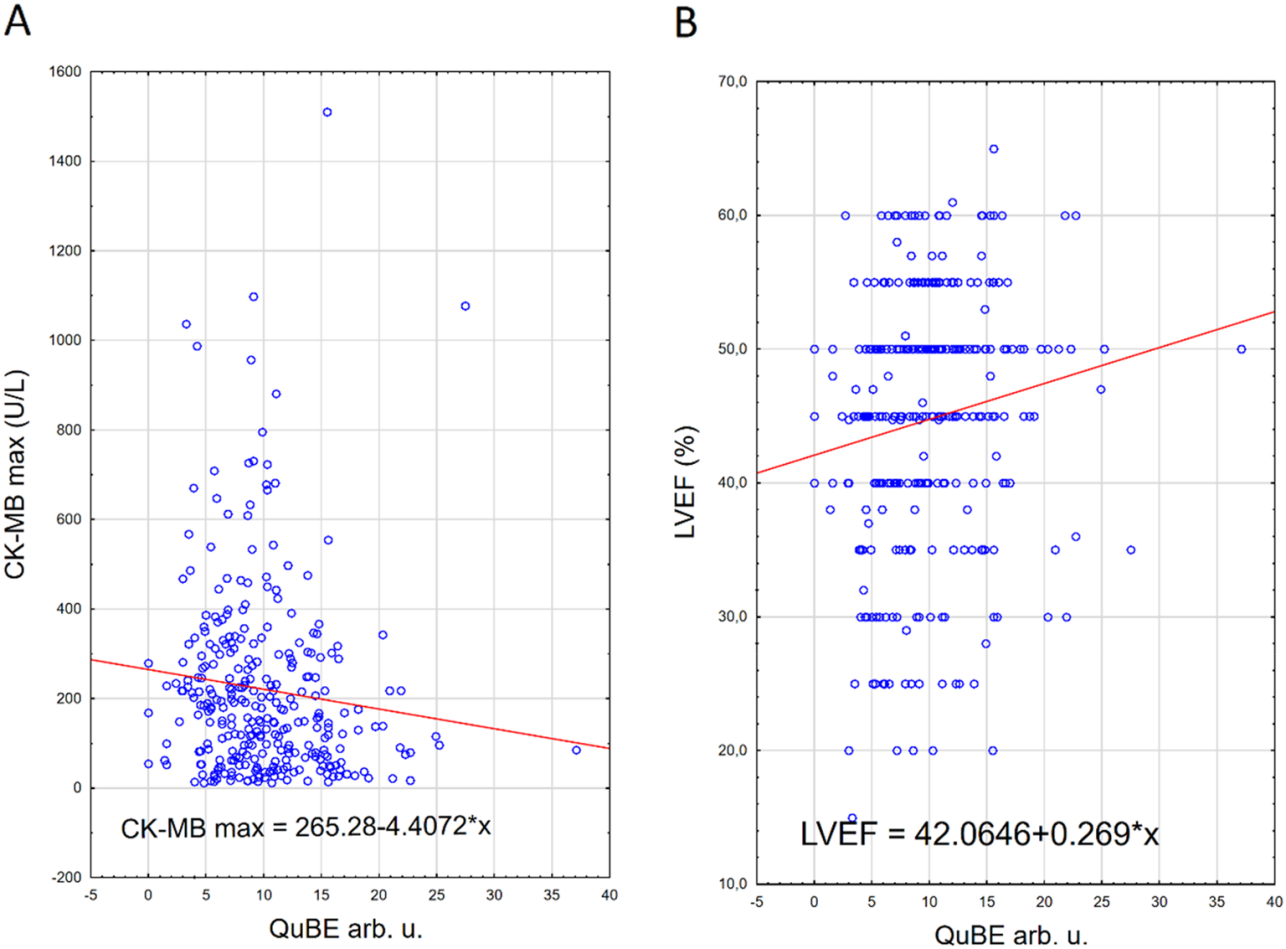
Graphical presentation of the relationship between peak CK-MB activity and QuBE (**A**) as well as LVEF and QuBE (**B**) in nondiabetic patients.

The interrelationship between QuBE, peak CK-MB, and LVEF in patients with T2DM is depicted in Fig. 1. The lowest mean LVEF values are seen in patients with the lowest QuBE and highest maximal CK-MB activity. Similar trends can be seen in patients without T2DM in Fig. 2.

## Discussion

In this ancillary analysis of an observational cohort study of T2DM patients with STEMI undergoing pPCI, we found that diminished left ventricular perfusion as quantified by QuBE was associated with significantly larger enzymatic infarct size and lower LVEF in both patients with T2DM and these without it, regardless of factors such as epicardial blood flow (measured by cTFC) or MI location. Second, older patients with T2DM had worse myocardial perfusion, greater peak CK-MB activity, and lower LVEF. These observations provide supplementary evidence to support our prior clinical observations that T2DM and diminished myocardial perfusion increase the risk of HF and/or all-cause mortality during 6-year follow up^15^.

In this study, QuBE correlated with LVEF, a well-established marker for prognosis of outcomes of patients after pPCI consistent with our previous finding that diminished myocardial perfusion assessed by QuBE was lower in patients with T2DM and was associated with higher long-term risk of HF and/or all-cause mortality. Therefore, QUBE could potentially be useful in risk assessment of patients with T2DM after pPCI due to STEMI.

Associations between infarct size, LV function and myocardial perfusion have been previously studied in patients with acute MI treated with pPCI using a wide variety of diagnostic modalities. Studies based on cardiac magnetic resonance (CMR) measurements showed that infarct size correlates with LVEF and subjects with microvascular obstruction had larger infarct size^17^. Similarly, infarct size as assessed by Single Photon Emission Computed Tomography (SPECT) closely correlates with LVEF^20^. Recently published study by Assante et al.^21^ provides the evidence for increased cardiovascular risk in diabetic patients with impaired myocardial perfusion assessed with positron emission tomography.

Limited data exist regarding enzymatic method estimating infarct size and measures of myocardial perfusion based on angiography. Consistent with the current study, Henriques et al.^22^ demonstrated that patients with worse myocardial perfusion had larger enzymatic infarct size and lower LVEF; however, Henriques et al. graded myocardial perfusion as a categorical variable using Myocardial Blush Grade (MBG), hence no detailed relationship between myocardial perfusion and infarct size was described. Also, infarct size estimated by release of CK-MB was larger when myocardial perfusion measured by MBG was worse, although again, conducted analysis considered perfusion as a categorical variable^23^.

There are other methods such as contrast echocardiography^18^ or magnetic resonance imaging^24^ used to evaluate myocardial perfusion, albeit not routinely, because of cost, limited access and equipment required. In contrast, QuBE can be assessed immediately after pPCI procedure with little effort. Another advantage of QuBE is the limited inter- and intra-observer variability^18,19^ as well as its continuous data output, in contrast to MBG and TIMI Myocardial Perfusion Grade (TMPG) which are graded visually as discrete values.

Few studies have addressed whether microvascular obstruction of myocardium is more extensive in patients with diabetes and STEMI and how it impacts their clinical outcomes. Two studies^11,13^ showed that patients with T2DM are more likely to have reduced myocardial perfusion, but others have presented opposing results^25^. Notably, these trials utilized different methods for myocardial perfusion assessment, that is, MBG and STR (ST segment resolution) in the studies that showed significant associations^11,13^ or the use of TMPG in study that did not^25^. Of note, there was no correlation between angiographic MBG and method based on contrast echocardiography TMPG for myocardial perfusion assessment^26^. In the current study, the patients with T2DM had lower myocardial perfusion measured by QuBE.

Although patients with T2DM had numerically lower CK-MB activities, those with poor myocardial perfusion (QuBE below median) had the greatest CK-MB activities in the study. Prior reports evaluating whether a relationship between diabetes and CK exists in a population of patients with acute MI had opposing results. For example, post-hoc analyses of the SAVE and CORE trials demonstrated significantly lower peak CK (all isozymes) when diabetes was present^27,28^. A case control study reported increased peak CK and CK-MB in patients with diabetes^29^. This discrepancy may be explained partly by differing baseline characteristics of patients between studies and poor characterization of study population in the observational study, whereby many potential confounders were not controlled for.

Another challenge is to establish whether worse outcomes associated with diabetes result from more extensive myocardial injury as compared to nondiabetics. Several studies^11,28,30–35^ analyzed infarct size, LVEF and other myocardial damage markers obtained by means of different imaging modalities (CMR, SPECT, echocardiography, or angiography). Although most studies reported the lack of diabetes impact on infarct size and LVEF^30–35^, there are also studies with conflicting results. For example, Marso et al.^11^ found significantly larger infarct size in patients with diabetes, possibly caused by worse myocardial perfusion in this group. A post-hoc analysis of the VALIANT trial^34^ did not reveal differences in regard to LVEF between patients with and without diabetes; however, an analysis of patient subgroups stratified by LVEF and diabetes in this study showed a reduced risk of adverse events (death or HF hospitalizations) with increasing LVEF although the magnitude of this risk reduction was smaller among patients with diabetes. A sub-study of the CORE trial showed that patients with diabetes and STEMI had larger infarct size and lower LVEF, but this alone cannot fully explain the much higher mortality of patients with diabetes^28^. Interestingly, Zia et al. showed increased myocardial oedema^32^, advocating another possible mechanism causing worse outcomes in diabetic STEMI patients.

While the aforementioned studies utilized different treatment regimens and modalities for assessment of functional parameters, the associations between parameters of myocardial perfusion, infarct size, left systolic function and DM were nonexistent or modest, but worse long-term clinical outcomes (MACE (major adverse cardiovascular events) or mortality) for patients with diabetes were consistent across all studies, ranging from approximately 40% to eightfold increased risk^11,28,30,31,33–35^.

### Limitations

The present study has several limitations. These include a relatively small sample size, and recruitment from one center in a retrospective manner. It has to be noted, that assessment of microvascular perfusion based on angiography is much less precise compared to other modalities, such as CMR. Consequently, it has been shown that microvascular obstruction seen in CMR is better at predicting outcomes than MBG^36^. Moreover, one must bear in mind that a non-standard method of QuBE measurement has been applied in this analysis. Additionally, since the time of patient recruitment, changes have emerged in the therapy of diabetic STEMI patients, including new hypoglycemic agents such as sodium-glucose co-transporter 2 inhibitors, and glucagon-like peptide-1 receptor agonists, which have cardioprotective effects. For example, liraglutide and canagliflozin administration significantly reduce infarct size in animal models^37,38^ and exenatide reduces infarct size in patients treated with pPCI due to STEMI^39^. Furthermore, advancements in pPCI are evident, including drug-eluting stents, reduced use of adjunctive thrombectomy and the introduction of new antiplatelet drugs.

## Conclusions

Diminished myocardial perfusion assessed by QuBE predicts significantly larger enzymatic infarct size and lower LVEF among patients with STEMI treated with pPCI, regardless of diabetes status.

## Data Availability

The datasets used and analysed during the current study are available from the corresponding author on reasonable request.

## Abbreviations

T2DM: Type 2 diabetes mellitus
HF: Heart failure
pPCI: Primary percutaneous coronary intervention
STEMI: ST elevation myocardial infarction
LVEF: Left ventricular ejection fraction
QUBE: Quantitative Myocardial Blush Evaluator
CK: Creatine kinase
CK-MB: Creatine kinase myocardial band
AUC: Area under the curve
cTFC: Corrected TIMI frame count
MI: Myocardial infarction
LV: Left ventricle
CMR: Cardiac magnetic resonance
SPECT: Single photon emission computed tomography
MBG: Myocardial blush grade
TMPG: TIMI myocardial perfusion grade
STR: ST segment resolution
MACE: Major adverse cardiovascular events
